# Largest Lyapunov Exponent Optimization for Control of a Bionic-Hand: A Brain Computer Interface Study

**DOI:** 10.3389/fresc.2021.802070

**Published:** 2022-02-11

**Authors:** Amin Hekmatmanesh, Huapeng Wu, Heikki Handroos

**Affiliations:** Laboratory of Intelligent Machines, LUT University, Lappeenranta, Finland

**Keywords:** brain computer interface, pattern recognition, EEG, largest Lyapunov exponent, imaginary pattern, optimization

## Abstract

This paper introduces a brain control bionic-hand, and several methods have been developed for predicting and quantifying the behavior of a non-linear system such as a brain. Non-invasive investigations on the brain were conducted by means of electroencephalograph (EEG) signal oscillations. One of the prominent concepts necessary to understand EEG signals is the chaotic concept named the fractal dimension and the largest Lyapunov exponent (LLE). Specifically, the LLE algorithm called the chaotic quantifier method has been employed to compute the complexity of a system. The LLE helps us to understand how the complexity of the brain changes while making a decision to close and open a fist. The LLE has been used for a long time, but here we optimize the traditional LLE algorithm to attain higher accuracy and precision for controlling a bionic hand. In the current study, the main constant input parameters of the LLE, named the false nearest neighbor and mutual information, are parameterized and then optimized by means of the Water Drop (WD) and Chaotic Tug of War (CTW) optimizers. The optimized LLE is then employed to identify imaginary movement patterns from the EEG signals for control of a bionic hand. The experiment includes 21 subjects for recording imaginary patterns. The results illustrated that the CTW solution achieved a higher average accuracy rate of 72.31% in comparison to the traditional LLE and optimized LLE by using a WD optimizer. The study concluded that the traditional LLE required enhancement using optimization methods. In addition, the CTW approximation method has the potential for more efficient solutions in comparison to the WD method.

## 1. Introduction

Recently, the integration of artificial intelligence (AI) techniques with wearable robots has been used in human applications, such as bionic hands and prosthetic orthosis. In our investigation, we used electroencephalograph (EEG) signal processing algorithms for the control of a bionic hand as a new design. One critical part of AI methods in this case is to develop methods for extracting discriminative patterns from the EEG signals which are relative to the desired actions. Here, we focused on an automatic identification method based on the largest Lyapunov exponent (LLE) chaotic feature to control a bionic hand robot. The principal of the control involved training our classifier regarding the EEG imagery patterns of a human hand which included opening the hand and making a fist.

Several features of wavelets (1), common spatial patterns (CSP) (2), and chaotic features (3) have been implemented for identifying imaginary movement patterns. Each of the above-mentioned features was used to follow at least one specific characteristic of imaginary movement patterns. For example, in wavelet-based methods (4), a predefined similar pattern to the target pattern, named a mother wavelet, is introduced to the wavelet packet algorithm. Then, the algorithm decomposes the signal into sub-frequencies and searches for similar patterns to the mother wavelet pattern. The wavelet search is based on comparing the different scales of the introduced mother wavelet by simultaneously shifting through time and the input signal. Depending on the complexity of the signal and the introduced mother wavelet, the obtained results are most significant, but a critical limitation exists: the computations are very time consuming. Therefore, the wavelets are not suitable the real-time systems, specifically brain computer interface (BCI) applications. Another, impressive computational feature CSPs which have obtained significant results, but a critical limitation of binary classification still exists. In our previous study, different classifiers with different versions of CSP algorithms was used and considered comprehensively. The importance of CSP features becomes apparent when two classes of identification are required. These methods are applicable for multi-class identification, but a high level of error rate were obtained. The other useful feature is LLE (5), which is widely used in different fields of studies such as schizophrenia (6, 7), sleep EEG processing and memory investigations, BCI applications for control of remote vehicles (4), as well as in bionic hands, and for the prediction of epilepsy seizure attacks (8, 9).

Largest Lyapunov exponent is a chaotic quantifier that gives system information if the system state moves toward chaos or stays in a stable condition. In our previous studies (4), we used the LLE to measure the complexity of brain signals to consider memory consolidation and learning (10), and the prediction of the onset of the subject's intention to move the right hand (11) to control a mobile vehicle (12). After several studies on the LLE algorithm, it has been shown that the limitations of the LLE such as being intensively dependent on the initial values and length of signal in the phase space play important roles in the accuracy of the results. Therefore, optimizing the initial LLE values by using evolutionary algorithms would be interesting.

Evolutionary algorithms have been widely used in solutions proposed for improving identification algorithms. For example, particle swarm optimization has been used for optimizing a neural network to extract EEG features (13). In another study (14), a binary flower pollination evolutionary algorithm was employed to identify EEG patterns and select the EEG channels for a feature extraction procedure. In another study (15), a Tug of War (TW) Optimizer was first introduced a solution was identified for different engineering problems such as optimizing the design of castellated beams (16). With respect to the results of various benchmarking optimization problems, Tug of War Optimizer would be a strong optimization method for increasing the identification performance.

Our contribution in the present study is employing colored pictures of an opening hand and making a fist to generate stronger ERD/ERS patterns for an artificial intelligence algorithm. The second part of the contribution is implementing an Optimized LLE (OLLE) algorithm by making use of the Water Drop (WD) and Chaotic Tug of War (CTW) optimizers (17) to identify two classes of opening a right hand and making a fist. To evaluate the efficiency of the OLLE and traditional LLE [the EEL introduced by Wolf (18)], imaginary features in the EEG were extracted and then employed for controlling a bionic hand to make a fist and to open the hand. In the algorithm, the investigation on the trajectories of the traditional LLE and OLLE were performed by illustrating the effects of initial values on the behavior of a chaotic system by using multiple figures. In the figures, it is also demonstrated how the separability of trajectories is applicable. The computed features are then classified by using our previous implemented classifier, named the Soft Margin Support Vector Machine with the Generalized Radial Basis Function (SMSVM-GRBF) (19). The rest of the paper is presented as follows: Section 2 explains the experimental setup for recording EEG; Section 3 explains the LLE feature extraction and optimization computations; Section 4 presents the obtained results; Section 5 is the discussion part; and Section 6 presents the conclusion.

## 2. Experimental Setup

**Figure 1:**
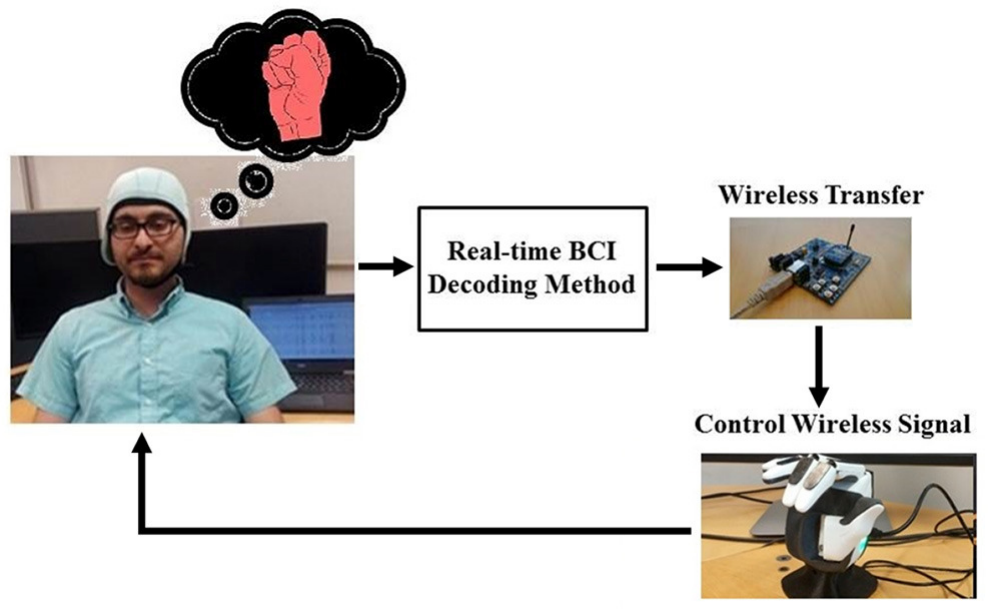
The experimental procedure and the experimental setup for controlling a bionic hand.

For recording EEG signals, 21 subjects participated in an experimental imaginary task. In the task pictures of making a right hand fist and open hand were displayed in the following sequence: (1) a black screen with a cross at the center; (2) a sketch of an open right hand or making a fist, which was colored in yellow and red, selectively; (3) a random resting period; and the cycle was then repeated. The experimental setup for the control of the bionic hand is presented in Figure 1. In the next step, we explain how the optimization was applied to update the LLE features. For the real-time experiment, five subjects participated for control of a bionic-hand, named Brunel Hand version 1.0 (https://openbionicslabs.com/shop/brunel-hand). For the experiment, we asked the participants for their informed consent and consult an ethics committee.

## 3. Methods

**Figure 2:**
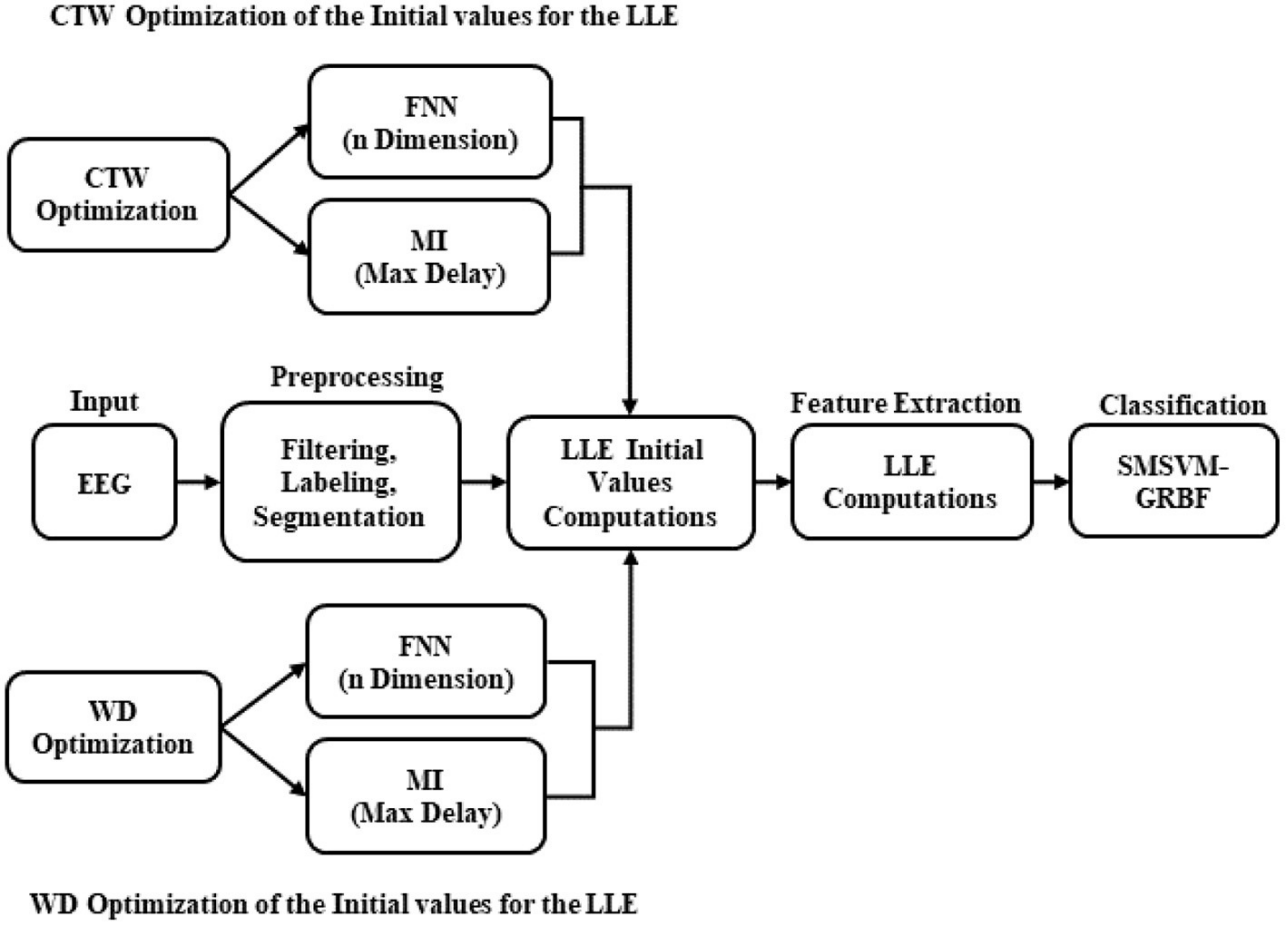
The algorithm schematic for identifying the ERD/ERS patterns.

In order to identify the ERD/ERS patterns, several steps were required to be applied, which are illustrated in Figure 2. In the initial steps, pre-processing is used to prepare the data for the LLE computations. The principal for computing the LLE features involves reconstructing a phase space based on the input values. According to Taken's theorem (20), the phase space is reconstructable using two delayed EEG signals. Afterward, the initial values in the functions of the LLE, named MI and FNN, are defined as free parameters. In our computations, the aim is to parameterize the constant values of the MI (maximum delay) and FNN (maximum embedding dimension) by means of CTW and WD algorithms. For this purpose, first, the LLE proposed by Wolf (18) needs to be implemented, which is based on the traditional MI and FNN as follows:

### 3.1. Pre-processing

During the data recording, several triggers (markers) have been sent to the data to mark the location of displaying images. Therefore, the EEG data was segmented with the interval of 200 ms before displaying images to 2,500 ms after displaying images. Afterward, the segments are sorted in a matrix and passed through a six order band pass Butterworth filter with 8–15 Hz edges. The prepared data is then employed for the feature extraction which is explained in the next part.

During the data recording based on the experimental task in Section 2, several triggers have been used automatically to mark the location of each step. Therefore, the EEG data was segmented with the interval of 200 ms before displaying picture's trigger to 2,500 ms after displaying the pictures. Afterward, the segments are sorted in a matrix and passed through a six order band pass Butterworth filter with 8–15 Hz edges. The prepared data is then employed for feature extraction in the next parts.

### 3.2. Mutual Information (MI)

To reconstruct a phase space, two delays of an input signal need to be computed, named time lag (ζ). It is critical in MI computations to select the length of the time lag. MI is a method to compute the lag with the principal of information between mutual time intervals *x*_*t*_ and *x*_*t*+ζ_. The lag ζ is computed by using the MI approach as follows:


(1) $$\begin{array}{l} MI\left(\zeta \right)=\overset{j}{\underset{i=1}{\sum }}\overset{j}{\underset{s=1}{\sum }}{Pr}_{i,s}\left(\zeta \right)\log_{2}\left(\frac{{Pr}_{i,s}\left(\zeta \right)}{{Pr}_{i}{Pr}_{s}}\right), \end{array}$$

where, *i* and *s* are the interval indexes for *x*_*t*_ and *x*_*t*+ζ_, respectively. Also, Pr_*i*_ and Pr_*s*_ are existing probabilities of *x*_*t*_ values in *i*th and *s*th, respectively. In the computations, it is important to limit the maximum lag to control the MI computations. In our previous studies, we set the maximum lag at 10 experimentally. In our algorithm, the maximum lag is parameterized using WD and CTW optimizers after computing the FNN.

### 3.3. False Nearest Neighbor (FNN)

Embedding dimension (*n*) is the next essential parameter for reconstructing a phase space. Dimension of a phase space dimension is obtained using the FNN algorithm, $x_{j}={\left[x_{s},x_{s+\zeta },x_{s+2\zeta },...,x_{s+\left(n-1\right)\zeta }\right]}^{\zeta },s=1,...,N$ and then used consistently for the rest of computations. Using the obtained MI and FNN values with the above-mentioned formulations, an attractor in the phase space is created with a no-intersection assumption. Generally, attractors are introduced as a trajectory of a system that tends to grow in the phase space. In order to satisfy zero intersection in the attractors, the dimension of phase space dimension needed to be increased until no intersection was counted. Therefore, FNN is the number of intersections in an attractor, which is used to increase the phase space dimension (21). In the computations, it is important to set a maximum dimension to limit the FNN computations while increasing the attractor dimensions in the phase space. Regarding our previous studies, the maximum embedding dimension for the FNN computation was set at three experimentally, in which we used it as the second free parameter in WD and CTUG optimizers to find the optimum values.

### 3.4. Largest Lyapunov Exponent

One method for quantifying a non-linear system is the Lyapunov exponent which was introduced by Wolf (18). The Lyapunov exponent is defined as the average value of exponential divergence along the grew trajectory for individual segments. The Lyapunov exponent (γ) outcome has three following conditions: 1- γ>0 means the system is in a chaotic situation, 2- γ = 0 means the system is in a limit cycle, and 3- γ <0 means the system is in a stable situation.

In order to obtain the LLE (γ_1_) feature, the divergence of the trajectory is applied on the pair neighbor trajectories in the reconstructed phase space by using $Q\left(t\right)=Ee^{\gamma_{1}t}$ at time *t* and an initial separation *E*. Next, between pairs of individual neighbors, logarithmic distance (*Q*_*j*_(*m*)) is computed by ln(*Q*_*j*_(*m*))≈γ_1_(*m*.Δ*t*)+ln (*E*_*j*_), where *m* is the counter of the pairs of neighbors. In the algorithm, the LLE is the maximum exponential slope for the segments calculated as follow (21):


(2) $$\begin{array}{l} LLE\left(i\right)=\frac{1}{\Delta t}\times \frac{1}{j}\overset{j}{\underset{1}{\sum }}\ln\;\left[Q_{j}\left(i\right)\right]. \end{array}$$

The obtained LLE features for the subjects are then optimized using the WD and CTW algorithms.

### 3.5. WD Algorithm

An intelligent WD concept is a swarm-based optimization algorithm, which is based on the action and reaction between water and soil in a river when it flows (22). The procedure of water movements in a river is flowing from one location to another location to find an optimum path to reach into a sea or lake. Therefore, the property of water movements and changing directions means the WD algorithm is an efficient optimizer for a discrete finite length system such as an EEG signal.

**Figure 3:**
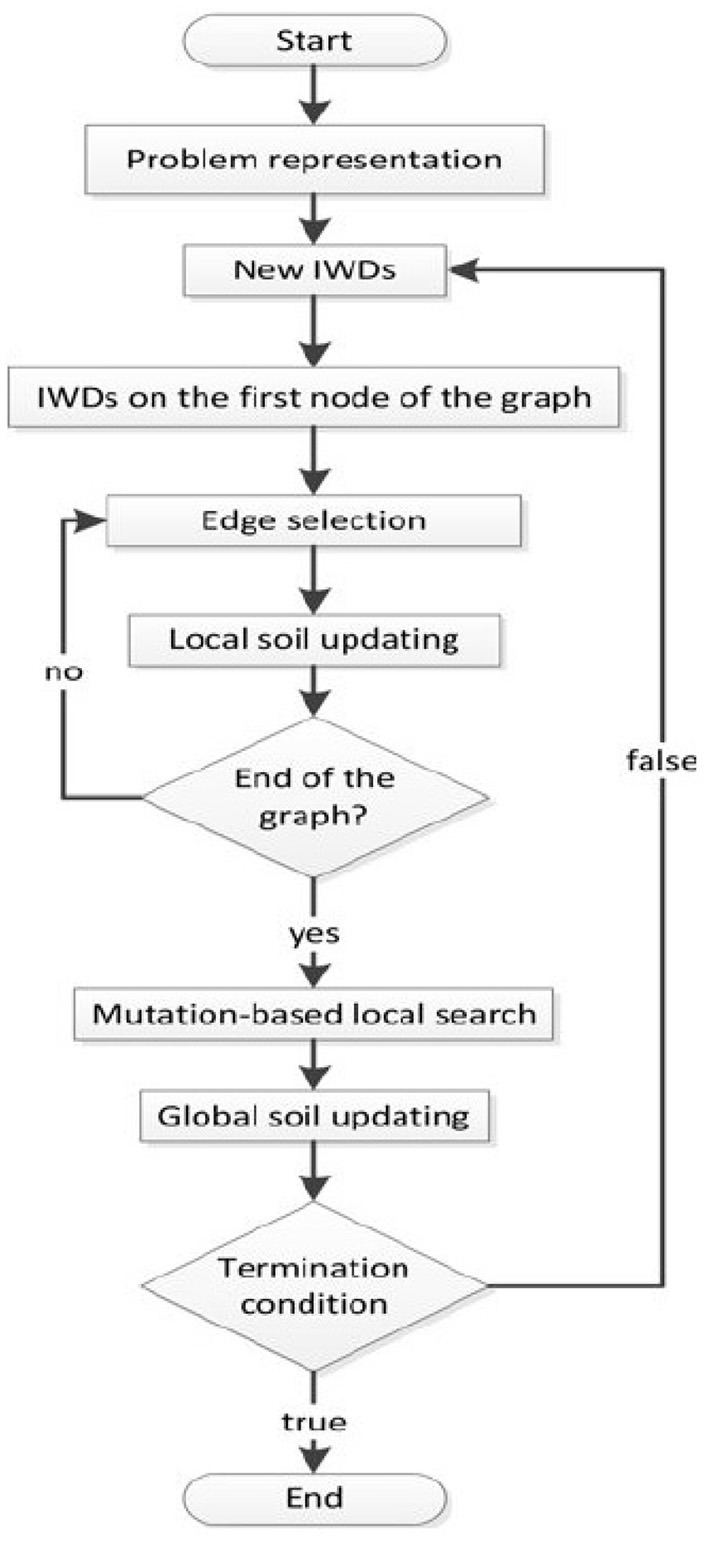
The graph shows the programming of the Water Drop (WD) optimization (23).

In the algorithm, two parameters are defined, namely soil and velocity. Soil defines the amount of soil that the river carries, and the velocity defines the velocity of the soil. The WD tends to move along a harder path (which means more soil flow) instead of an easier path (less soil flow). The algorithm concept is presenting a graph with an *N*, node set and an *E*, edge set. The above-mentioned graph means an environment that water flows on the edges of the environment. During the optimization, WD starts to construct a solution gradually by moving among the nodes until the final solution *T*^*WD*^ is found. Figure 3 shows the flowchart of the WD algorithm and the pseudo-programming tips are summarized as follows:

- Initializing the algorithm by forming a graph for searching (environment).
- Initializing soil and velocity by means of random values.
- WD distribution in the environment.
- Gradual solution reconstruction based on updating the soil and velocity.
- Local soil updating.
- Global soil updating.
- Best solution updating.
- Stop if the termination criteria are satisfied otherwise, go to step 2.

The details of mathematical formulations are presented (22, 23).

### 3.6. Chaotic Tug of War

Kaveh (16) introduced the TW optimization method on the concepts of interaction between two groups in a TW competition. This method has also been effectively used in our previous method in a BCI study (24). With respect to the principles, the algorithm is developed as follows:

In the first step, initial random values are selected to start the optimization, which is done randomly as follows:


(3) $$\begin{array}{l} X_{i}^{0}=L_{B}+rand*\left(U_{B}-L_{B}\right),\;i=1,\ldots ,N \end{array}$$

where $X_{i}^{0}$ is the initial value, *N* is the number of initial values, and *U*_*B*_ and *L*_*B*_ are the upper and lower limit of the search area, respectively. Then, each initial value is considered as a teams and weights are assigned to them for a TW competition as follows:


(4) $$\begin{array}{l} W_{i}=\frac{F_{i}-F_{worst}}{F_{best}-F_{worst}}+1,\;i=1,\ldots ,N \end{array}$$

where *W*_*i*_ and *F*_*i*_ are the weight and fitness values of the *i*th initial value, respectively. In each iteration, *F*_*best*_ and *F*_*worst*_ are the best and worst fitness functions between teams, respectively. Next, searching and updating values for the competition are performed as follows:


(5) $$\begin{array}{l} \begin{array}{l} \Delta X_{i}=\overset{N}{\underset{j=1}{\sum }}\Delta X_{ij},\\ \Delta X_{ij}=\frac{1}{2}a_{ij}\Delta t^{2}+\alpha \beta \left(U_{B}-L_{B}\right)*rand, \end{array} \end{array}$$

where Δ*X*_*ij*_ is the updating parameter for the competition between an *i*th team with *j*th team, β is a factor between (0, 1) and α is the proportional factor. The acceleration (*a*_*ij*_) of the *i*th team in comparison with the *j*th team is computed as follows:


(6) $$\begin{array}{l} \begin{array}{l} {\;a}_{ij}=g_{ij}*\frac{F_{r,ij}}{W_{i}\mu },\\ {\;g}_{ij}=X_{j}-X_{i},\\ F_{r,ij}=F_{p,ij}-W_{i}\mu , \end{array} \end{array}$$

where *F*_*r, ij*_ and *g*_*ij*_ are the resultant forces affecting factors and the gravitational acceleration constant, respectively, and *F*_*p, ij*_ is the pulling force between teams with boundaries (*W*_*i*_μ, *W*_*j*_μ). The new teams as the next generation are computed as follows:


(7) $$\begin{array}{l} X_{i}^{new}=X_{i}^{old}+\Delta X_{i}. \end{array}$$

The final step is the termination rules based on the previous steps. If the searching procedure for selecting the teams achieves maximum accuracy or meets any of the stop criteria in the previous steps, then the algorithm returns the values, otherwise the algorithm continues searching by selecting new teams.

The optimization methods use random values for searching an area of numbers. Different methods proposed different solutions for converging the area of searching numbers with higher speed. Here, first, a random number is selected in Equation (5) and then the searching procedure continues by changing the searching direction using a defined fitness value. In the CTW optimization method, a chaotic map is employed to calculate the fitness factor. Therefore, Equation (5) is developed as follows:


(8) $$\begin{array}{l} \Delta X_{ij}=\frac{1}{2}a_{ij}\Delta t^{2}+\xi_{ij}*\left(U_{B}-L_{B}\right), \end{array}$$

where ξ_*ij*_ is the chaotic searching factor and computed as


(9) $$\begin{array}{l} \xi_{ij}=\Xi \left(F_{i}-F_{j}\right), \end{array}$$

where Ξ is the chaotic map. Well-known chaotic maps were used in our previous study (24). The concept of the chaotic maps and properties are presented in the next step.

#### 3.6.1. Chaotic Maps

Chaotic maps (Ξ_1_) are random based methods, which are employed in optimization approaches, specifically chaotic optimization. Chaotic searches are based on non-repetition and ergodicity, which are different from stochastic search methods, which are based on probabilities. The above-mentioned chaotic properties are an advantage for a full search with higher speed in comparison to stochastic searches. In chaos-based procedures, twelve non-invertible maps (Ξ_1_) are applied to generate chaotic sets of values to achieve a chaos goal, which means a complete search at high speed. More details of the twelve maps are available (25). In the next part, we consider the CTW validity.

#### 3.6.2. CTW Validation

In order to consider the validity of CTW against the basic TW algorithm, ten different chaotic benchmark functions were employed. The list of benchmark function formulas is introduced in our previous study (24). In short, if a new method is designed, it should be validated by using different validated benchmark functions, which are categorized into separable and non-separable generic groups. The implemented algorithm is applied on different chaotic maps, which are divided into separable and non-separable groups. In the CTW algorithm, the stop search criteria reaches the maximum number of evaluations of the cost function, which is set to 10,000.

The performance of CTW in comparison to other algorithms, such as the traditional TW algorithm, Genetic Algorithm (GA), and Particle Swarm (PS) optimizations are shown in Table 1. The obtained improvements based on the ten benchmark functions for the CTW against the traditional TW optimizer are obvious in Table 1. The extracted features are then classified by using our previous improved Support Vector Machine (SVM)-classifier, namely the SMSVM-GRBF (24).

**Table 1:** The results of Chaotic Tug of War (CTW) with different chaotic maps, Tug of War (TW), Genetic Algorithm (GA), and Particle Swarm (PS) (24).

**Algorithm**	**Maps**	***F*_1_**	***F*_2_**	***F*_3_**	***F*_4_**	***F*_5_**	***F*_6_**	***F*_7_**	***F*_8_**	***F*_9_**	***F*_10_**
**CTW**	Ξ_1_	0	0.0548	5.5E-06	0	0.0060	0.0642	4.8E-03	0.0742	0	8.8E-06
	Ξ_2_	0.0754	0	0.0597	0	7.5E-05	0.0694	0	6.4E-09	0.0030	0.0755
	Ξ_3_	0.878	4.8E-07	0.0070	0	0.0675	0	5.9E-08	0.0861	0.0732	0
	Ξ_4_	6.7E-09	0.0889	0.0046	0.0074	5.4E-06	0.0768	0.0819	0	0.0724	8.4E-05
	Ξ_5_	0	0.0778	0.0963	0.0043	7.6E-02	0.0716	6.6E-08	0	0.0073	0.0928
	Ξ_6_	4.4E-08	0.0744	0.0877	0.0048	0	7.7E-04	0.0766	0.0055	0.0433	6.2E-05
	Ξ_7_	0.0533	0	2.3E-01	0.0088	0.0812	0.0625	3.4E-05	0	0.716	0.0026
	Ξ_8_	6.9E-06	0.0466	0.0749	0	5.4E-03	8.9E-04	0.0032	0.0955	0.0043	6.7E-03
	Ξ_9_	0.0549	0.0645	0.0089	0	5.8E-06	0.0926	0.0025	4.4E-07	3.7E-04	0
	Ξ_10_	0.0866	6.1E-06	0.0755	0.0066	0	8.2E-06	0.0911	0.0028	0.0618	0
	Ξ_11_	0	4.5E-07	0.0743	0.0033	0.0621	5.1E-01	0.0477	0	6.2E-08	0.0027
	Ξ_12_	3.7E-04	0	0.0744	0.0053	0.0944	0	0.0046	8.7E-04	0.0655	0
**TW**	-	0	8.8E-02	0.0744	0.0834	4.8E-04	0.0522	0.0046	0.0876	0.0051	6.4E-09
**PS**	-	0.0499	0	2.6E-05	0.0621	3.7E-06	0.0082	0.0766	0	0.0839	7.3E-05
**GA**	-	0	7.2E-03	0.0974	0.0056	0	0.0566	8.1E-06	0.0814	0.0092	6.2E-03

### 3.7. Classification

The extracted features need to be classified by means of a classifier. In our previous study, we studied the efficiencies of the classifiers for a BCI study (19). Here, we employed the SMSVM-GRBF algorithm. The advantages of the SMSVM algorithm are that it enables the SVM to be optimized by free parameters in the regularization part and the GRBF has advantages in providing a Gaussian function with better coverage of the scattered features in the feature space by using three parameters, namely the width, shape, and center in a Gaussian shape.

For training, the SMSVM-GRBF model, the extracted features from 21 subjects were mixed and 70% of them were selected randomly for training the model. Then, 25% of the data were selected for testing, and the rest of the features (5%) was used for the validation.

## 4. Results

In the experiment, 21 subjects participated in the imaginary movement's task. Three different LLE-based algorithms were designed to identify the imaginary ERD/ERS patterns. Comparative Table 2 shows the accuracy results of traditional LLE, OLLE by using the CTW and DW optimizers for offline and real-time modes. Additionally, lag and dimensions are presented, which are the initial values for MI and FNN, respectively.

**Table 2:** The comparative accuracy results of traditional the largest Lyapunov exponent (LLE), optimized LLE (OLLE) using CTW and DW algorithms.

**-**	**Traditional** **LLE**	**OLLE with** **WD**	**OLLE wih** **CTW**
**Max selected lag**	10	91	96
**Max selected dimension**	3	53	45
**Offline average** **accuracy**	63.77%	68.36%	72.31%
**Offline average** **accuracy**	60.00%	60.00%	65.00%

**Figure 4:**
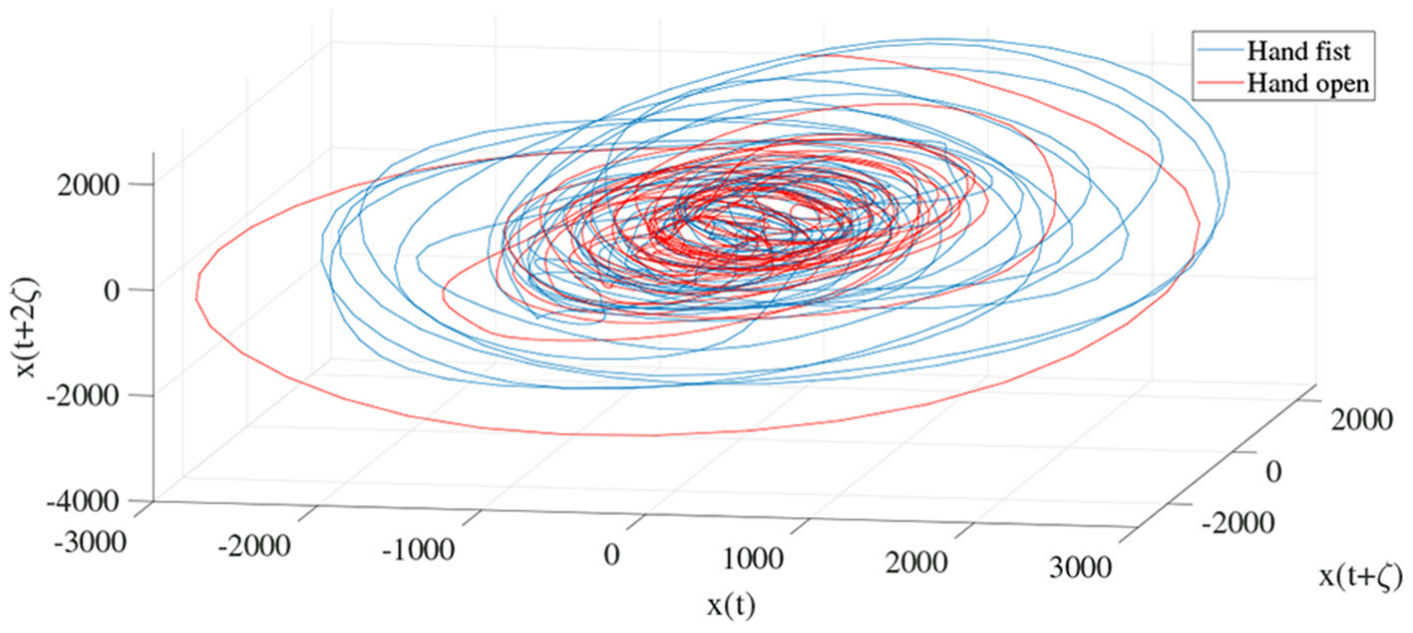
Trajectories of an open hand and making a fist in the reconstructed phase space for channel C3 (24).

**Figure 5:**
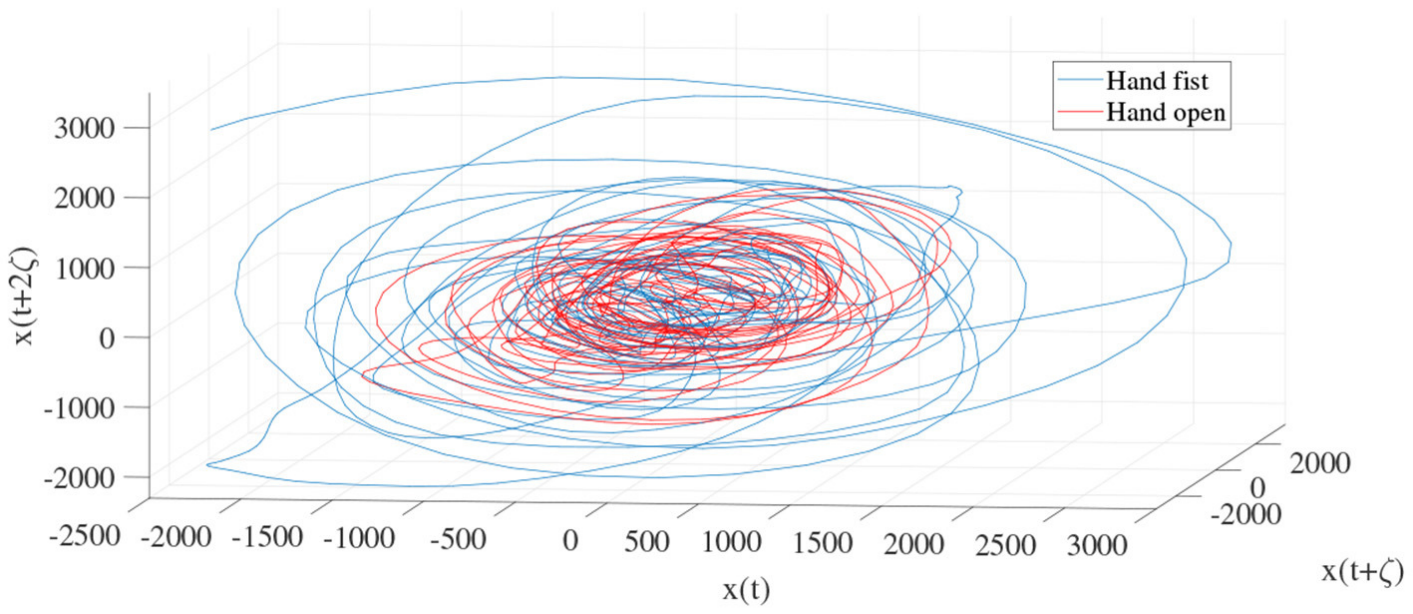
Trajectories of an open hand and making a fist in the reconstructed phase space for channel CP5 (24).

**Figure 6:**
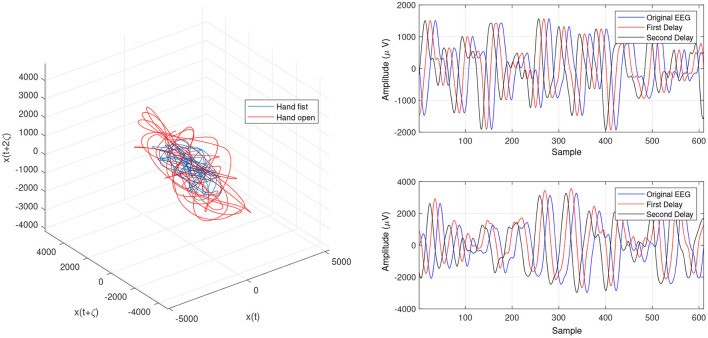
Trajectories of hand open and making a fist by using the optimized LLE (OLLE) in the reconstructed phase space based on the WD for channel C3.

**Figure 7:**
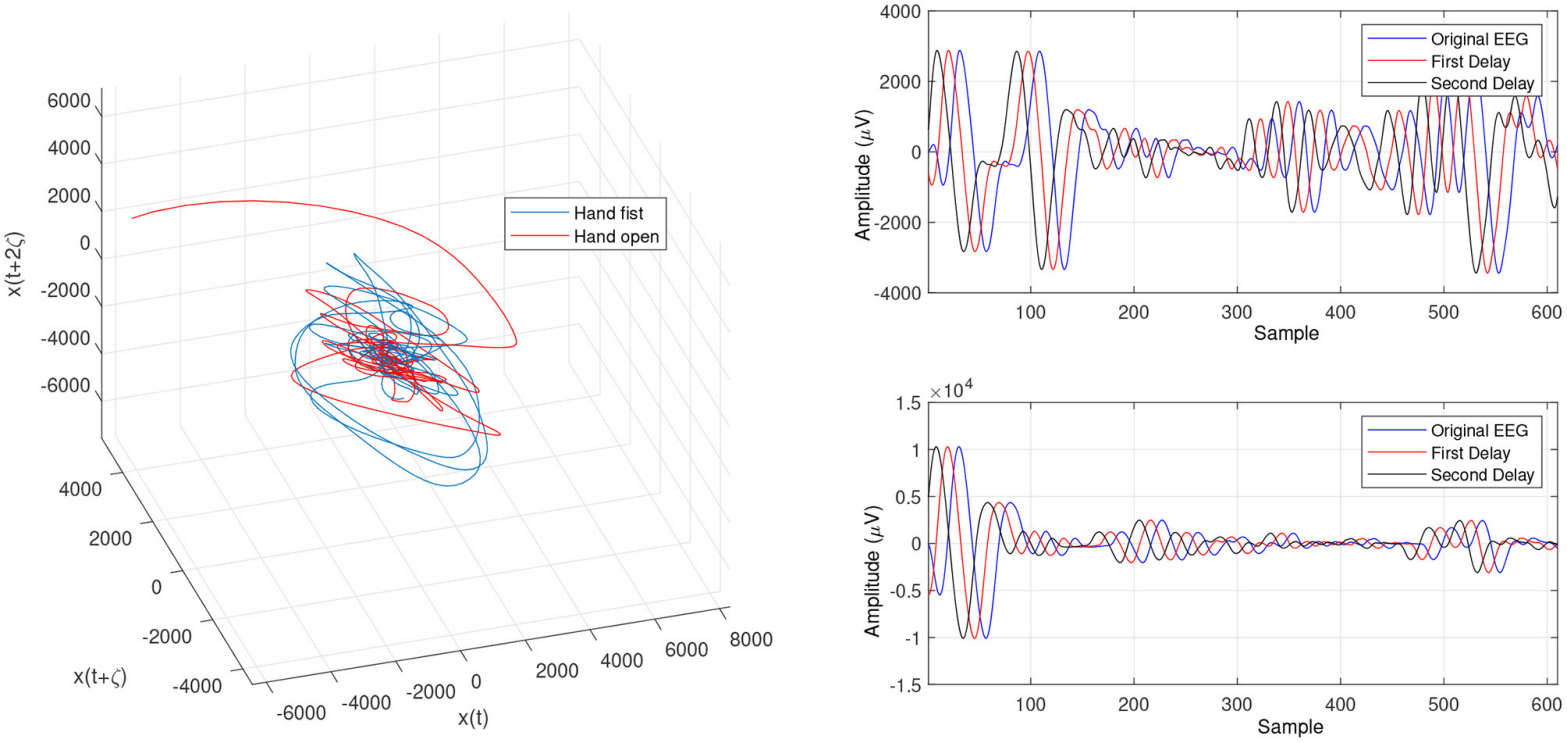
Trajectories of an open hand and making a fist using the OLLE in the reconstructed phase space based on the WD for channel CP5.

**Figure 8:**
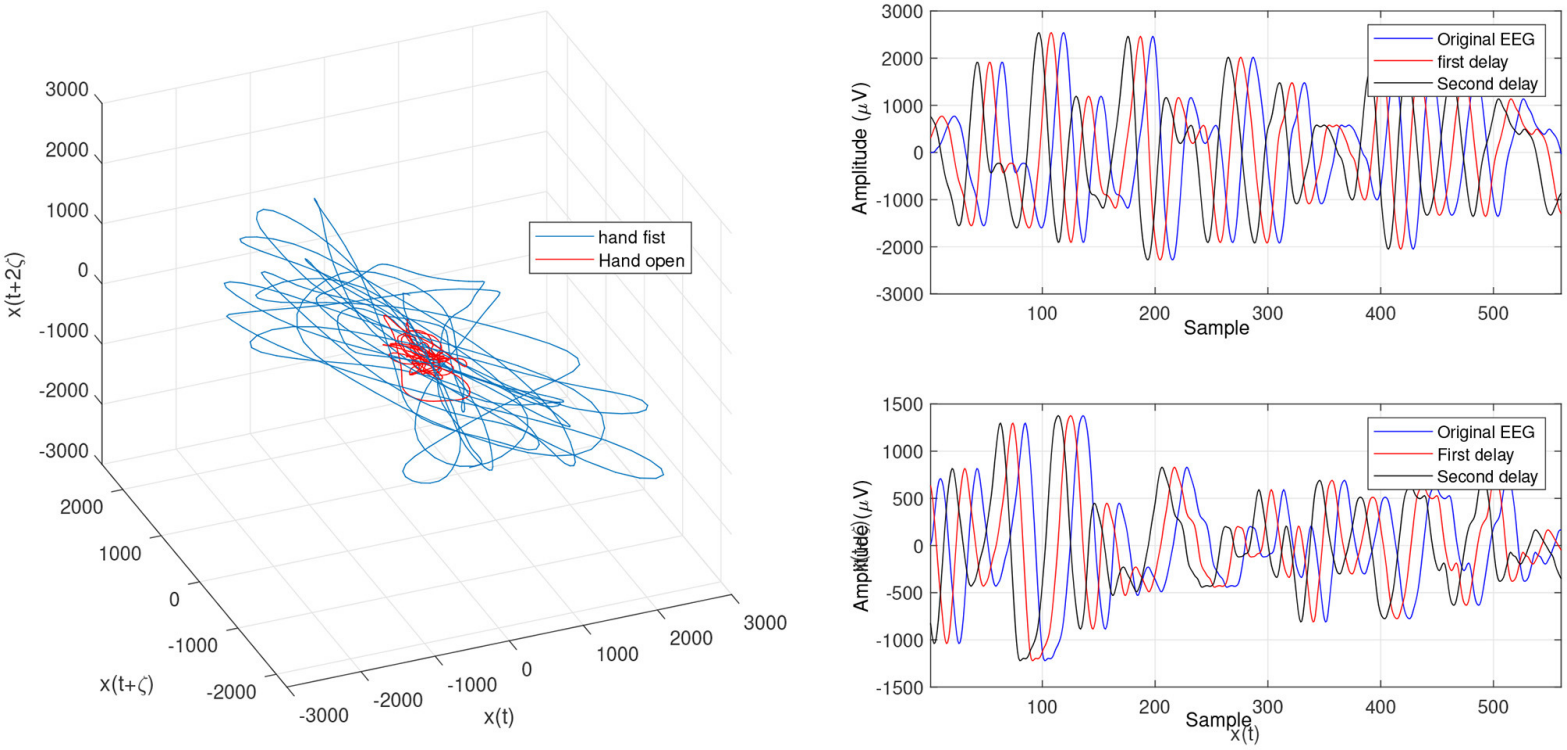
Trajectories of an open hand and making a fist by using OLLE in the reconstructed phase space based on the Chaotic Tug of War (CTW) for channel C3.

**Figure 9:**
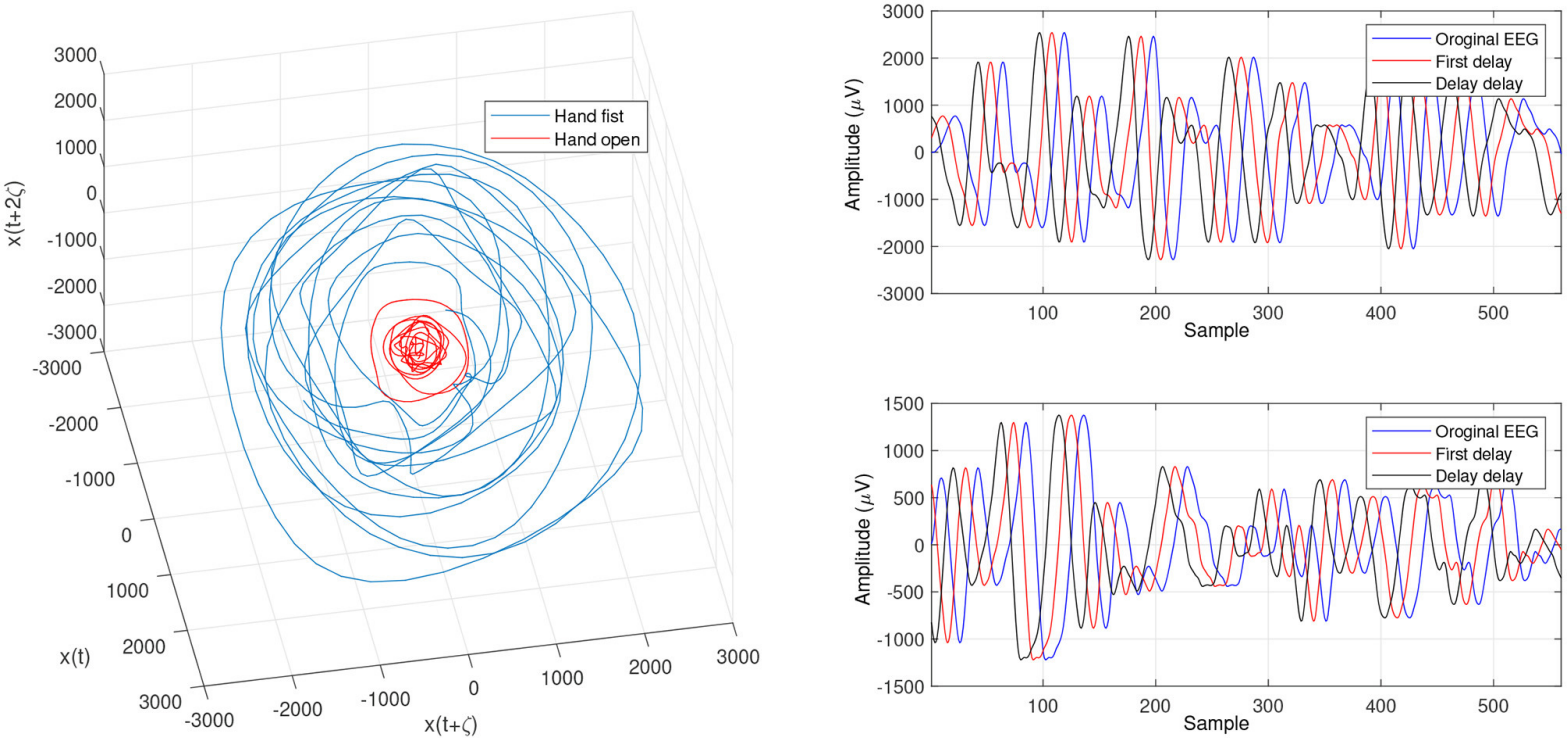
Changed projection of trajectories for an open hand and making a fist by using the OLLE in the reconstructed phase space based on the CTW for channel C3.

In order to determine the effects of the optimization algorithms on the LLE and phase space reconstruction, the figures of trajectories based on WD (**Figures 6**, **7**) and CTW (**Figures 8**, **9**) are illustrated. Additionally, the two delayed EEG signals for reconstructing the phase space in the figures are demonstrated and compared with the traditional LLE method in Figures 4 and 5.

## 5. Discussion

Regarding our previous studies (4), the most informative features for identifying the ERD/ERS patterns were extracted from the channels C3 and CP5 in the frequency range of 8–13 Hz. With respect to Taken's theory (20), to extract the LLE features a time lag and embedding dimension parameters need to be computed by means of MI and FNN approaches, respectively. The obtained MI and FNN are then employed to reconstruct a phase space. The MI is used to extract information between two interval points with the time lag τ. At the same time, the FNN is used to increase the embedding space dimension since false neighbors are segregated in the new trajectory in the reconstructed phase space. In the MI and FNN algorithms, it is essential to limit the maximum time lag and maximum embedding dimension in fixed terms of τ = 10 and *n* = 3 experimentally, respectively. The MI and FNN approximations have been developed in different studies and the criteria limitation still exists (21, 26–28).

In the present study, we focused on LLE optimizing methods using evolutionary algorithms to extract imaginary movement features for the control of a prosthetic hand. We worked on the main limitations of the LLE-based methods which are optimizing the initial LLE values. This provides an opportunity to control chaotic systems even though they are highly sensitive. The LLE has two initial values, named the FNN and MI, which are employed to reconstruct a phase space based on the two delayed input signals (Figures 6, 7, 9). In the LLE algorithm, the input EEG trajectories are calculated in a reconstructed phase space by means of mapping computations. The trajectories' properties are then used for computing the LLE (γ) feature, which has the three following conditions: (1) γ>0 means the system is in a chaos condition; (2) γ = 0 means the system is in a limit cycle condition; and (3) γ <0 means the system is in a stable condition. In one sense, the LLE is an average of exponential divergence rate along the trajectory in the initial points of the phase space. In the present study, we optimized the initial values in the FNN and MI by means of evolutionary algorithms, namely CTW and WD optimizers.

Currently, we employed the WD and CTW methods to approximate the criteria of FNN and MI in the LLE algorithm. The TW approximation is a competition between two teams that is based on the stochastic updating method to find the best solution. Our modified CTW has the same concept of competition between two teams with a significant difference in using chaotic maps instead of a stochastic approach. The advantage of chaotic maps is the properties of ergodicity and non-repeating behavior. On the other hand, the WD method concept is based on the parameters of soil and velocity that represent a population-based intelligence algorithm, where each drop is a solution that by sharing among drops the algorithm leads to a better solution.

To consider the proposed methods, two trajectories for the conditions of opening a hand and making a fist should be drawn in a reconstructed phase space based on the approximation of FNN and MI. To this end, two free parameters for an open hand and two free parameters for making a fist were defined. After the approximation computations, two trajectories calculated in the phase space with no intersection. The efficiency of the results was considered by means of the separability of trajectories, and the larger number of subjects whose features obtained significant and higher accuracies. Therefore, three sets of results relative to the traditional LLE, OLLE with the WD, and OLLE with the CTW are presented.

Figures 4 and 5 are the calculated trajectories for opening a hand and making a fist in the reconstructed phase space based on the traditional method. The phase space is reconstructed based on two delayed EEG signals and the LLE features with respect to the traditional initial consistent values in the FNN and MI computations. The discrimination of trajectories for the hand movements is challenging and the reported accuracy results showed a low accuracy rate of 63.77%. Optimizing the reconstructed phase space by using the WD approximation algorithm reduced the complexity of the trajectories in the phase space (Figures 6, 7) which provides an opportunity to increase the average accuracy result slightly (68.36%). The changes between traditional and optimized figures show the effects of the initial values on the features and classification results. In the algorithm, the approximated maximum lag for the MI and the approximated embedding dimension for the FNN were 91 (valuates between 2 to 91) and 53 (variates between 7 to 53), respectively. The second method for optimizing the reconstructed phase space was the CTW approximation algorithm. Figure 8 shows that the complexity of trajectories in the phase space is reduced significantly and the average accuracy result increased to 72.31%. In the algorithm, the approximated maximum lag for the MI and approximated embedding dimension for the FNN were 96 (valuates between 2 to 96) and 52 (variate between 2 to 52). The obtained results showed that the reconstructed phase space based on the traditional method and WD algorithms could not find the best lag and embedding dimension for the LLE computations. In order to consider the efficiency of the model in the real-time mode, the trained model was used to control a bionic hand. In the experiment, five subjects participated, and the best obtained accuracy result was obtained by using the LLE with the CTW optimization achieving 65.00% which was not significant. Additionally, the accuracy results in the real-time mode were insignificant.

To consider the meaningfulness of the obtained OLLE features for hand opening and making a fist, first, a paired *t*-test statistical analysis was applied. If the computed *t*-test was found to be meaningful (*P* > 0.05), we use the features for the classification. In the experiments, three insignificant subject features were found by using the OLLE with the WD algorithms, and one insignificant subject feature was found by using the OLLE with the CTW algorithm, while seven subjects features were revealed to be insignificant by using the traditional LLE. In order to evaluate the identification results, the SSVM-GRBF classifier trained 100 times and the average accuracy values are presented in Table 2. Regarding the obtained accuracy results in Equation 2, the CTW obtained the best average accuracy (72.31%) among the other methods and the traditional LLE algorithm obtained the lowest accuracy (63.77%). It is supposed that the ergodicity and non-repetition in the CTW led to finding better solutions at a higher speed in comparison to the WD algorithm (68.36%).

The disadvantage of the evolutionary approximation is the obtained approximated values in each train cycle change. The reason for this is that the initial values for searching an area are selected randomly. The advantage of the CTW methods is that there is no limitation for optimizing a set of parameters at the same time. Here, we could have optimized four parameters, which means that in the search area the four selected parameters are the best solutions for higher accuracy. Additionally, in the search area, the algorithm proposed different solutions for the four parameters that achieved the same accuracy.

## 6. Conclusion

In the present study, different methods based on the LLE were implemented to identify imaginary patterns and control of a bionic hand. In the procedure, a phase space needed to be reconstructed using the FNN and MI approaches, which are highly sensitive to the initial values named the maximum time lag and maximum embedding dimension, respectively. First, a traditional LLE was implemented and showed how the trajectories in the reconstructed phase space are highly complicated. The initial values were then approximated using the WD and TUW methods. The obtained results based on the new initial values were considered and compared. Different plots were employed to show the effects of changing the initial values based on the approximated algorithms. It showed that the CTW optimization algorithm reach the highest accuracy rate of 72.31% in comparison to the WD optimizer and traditional LLE method.

## Data Availability Statement

The original contributions presented in the study are included in the article/supplementary material, further inquiries can be directed to the corresponding author.

## Author Contributions

AH has the main contribution for EEG signal processing, analyzing, and preparing the paper.

## Conflict of Interest

The authors declare that the research was conducted in the absence of any commercial or financial relationships that could be construed as a potential conflict of interest.

## Publisher's Note

All claims expressed in this article are solely those of the authors and do not necessarily represent those of their affiliated organizations, or those of the publisher, the editors and the reviewers. Any product that may be evaluated in this article, or claim that may be made by its manufacturer, is not guaranteed or endorsed by the publisher.
